# Correction: Sugimoto et al. Trends in the Prevalence and Progression of Diabetic Retinopathy Associated with Hyperglycemic Disorders during Pregnancy in Japan. *J. Clin. Med.* 2022, *11*, 165

**DOI:** 10.3390/jcm11102789

**Published:** 2022-05-16

**Authors:** Masahiko Sugimoto, Kohei Sampa, Hideyuki Tsukitome, Kumiko Kato, Hisashi Matsubara, Shin Asami, Kaori Sekimoto, Shigehiko Kitano, Shigeo Yoshida, Yoshihiro Takamura, Takao Hirano, Toshinori Murata, Miho Shimizu, Takamasa Kinoshita, Sentaro Kusuhara, Osamu Sawada, Masahito Ohji, Rina Yoshikawa, Kazuhiro Kimura, Hiroto Ishikawa, Fumi Gomi, Hiroto Terasaki, Mineo Kondo, Tomoaki Ikeda, on behalf of the Writing Committee of Japan-Clinical Retina STudy Group (J-CREST)

**Affiliations:** 1 Department of Ophthalmology, Graduate School of Medicine, Mie University, Tsu 514-8507, Japan; k.sampa0525@gmail.com (K.S.); dyrkn618@yahoo.co.jp (H.T.); cqw14171jp@yahoo.co.jp (K.K.); hmatsu@clin.medic.mie-u.ac.jp (H.M.); mineo@clin.medic.mie-u.ac.jp (M.K.); 2 Graduate School of Medicine, Faculty of Medicine, Mie University, Tsu 514-8507, Japan; 318002@m.mie-u.ac.jp; 3 Department of Ophthalmology, Diabetes Center, Tokyo Women’s Medical University, Tokyo 162-8666, Japan; sekimoto.dmc@twmu.ac.jp (K.S.); ge2s-ktn@asahi-net.or.jp (S.K.); 4 Department of Ophthalmology, Graduate School of Medicine, Kurume University, Kurume 830-0011, Japan; usyosi@gmail.com; 5 Department of Ophthalmology, School of Medical Sciences, University of Fukui, Yoshida 910-1193, Japan; ytakamura@hotmail.com; 6 Department of Ophthalmology, School of Medicine, Shinshu University, Matsumoto 390-8621, Japan; takaoh@shinshu-u.ac.jp (T.H.); murata@shinshu-u.ac.jp (T.M.); 7 Department of Ophthalmology, Sapporo City General Hospital, Sapporo 060-8604, Japan; miho.shimizu@doc.city.sapporo.jpn (M.S.); knst129@gmail.com (T.K.); 8 Division of Ophthalmology, Department of Surgery, Graduate School of Medicine, Kobe University, Kobe 650-0017, Japan; kusu@med.kobe-u.ac.jp; 9 Department of Ophthalmology, Shiga University of Medical Science, Otsu 520-2192, Japan; eye.sawada@gmail.com (O.S.); ohji@belle.shiga-med.ac.jp (M.O.); 10 Department of Ophthalmology, Graduate School of Medicine, Yamaguchi University, Ube 755-8505, Japan; rinayskw@yamaguchi-u.ac.jp (R.Y.); k.kimura@yamaguchi-u.ac.jp (K.K.); 11 Department of Ophthalmology, Hyogo College of Medicine, Nishinomiya 663-8501, Japan; ohmyeye@gmail.com (H.I.); fgomi@hyo-med.ac.jp (F.G.); 12 Department of Ophthalmology, Graduate School of Medical and Dental Sciences, Kagoshima University, Kagoshima 890-8520, Japan; teracchi16@yahoo.co.jp; 13 Department of Obstetrics and Gynecology, Graduate School of Medicine, Mie University, Tsu 514-8507, Japan; t-ikeda@clin.medic.mie-u.ac.jp

## Error in Table

In the original publication [1], due to an editorial office error, there were mistakes in: “Table 1. The prevalence of DR from the Japanese claim database cohort; Table 2. Complications during pregnancy from Japanese claim database cohort; Table 3. The distribution of DR stage from the multicenter cohort; Table 4. DR progression ratio from multicenter cohort.” as published. “The columns of the tables were divided by mistake”. The corrected “Table 1. The prevalence of DR from the Japanese claim database cohort; Table 2. Complications during pregnancy from Japanese claim database cohort; Table 3. The distribution of DR stage from the multicenter cohort; Table 4. DR progression ratio from multicenter cohort.” appear below. The authors apologize for any inconvenience caused and state that the scientific conclusions are unaffected. This correction was approved by the Academic Editor. The original publication has also been updated.

## Figures and Tables

**Table 1:** The prevalence of DR from the Japanese claim database cohort.

Total			DR (+)		DR (−)		Odds Ratio for DR between the Groups	*p*-Value
	N	Age (Years)	N (%)	Age (Years)	N (%)	Age (Years)		
**pexD**	1139	34.2 ± 4.6	73 (6.4)	34.4 ± 4.6	1066 (93.6)	34.2 ± 4.6		
**GDM**	4129	33.7 ± 4.5	61 (1.5)	35.4 ± 4.5	4068 (98.5)	33.7 ± 4.5		
**Total**	5268	33.8 ± 4.5	134 (2.5)	34.8 ± 4.5	5134 (97.5)	33.8 ± 4.5	4.6 (3.2–6.5) **	1.44 × 10^−15^

DR: diabetic retinopathy, GDM: gestational diabetes mellitus, pexD: pre-existing diabetes mellitus. Values are presented as the mean ± standard deviation. ** *p* < 0.01.

**Table 2:** Complications during pregnancy from Japanese claim database cohort.

	pexD		GDM		Odds Ratio (95% CI)	*p*-Value
	DR (+)	DR (−)	DR (+)	DR (−)		
**<General complications>**						
**Hypertension**	13 (11.7%)	98 (88.3%)	8 (2.1%)	367 (97.9%)	6.0 (2.5–15.0) **	9.78 × 10^−5^
**Renal failure**	9 (19.2)	38 (80.9)	1 (1.1)	95 (99.0)	22.5 (2.8–183.7) **	0.003
**Eclampsia**	0 (0)	0 (0)	0 (0)	13 (100)	Not available	Not available
**Caesarean section**	25 (11.7)	189 (88.3)	12 (1.5)	812 (98.5)	9.0 (4.4–18.1) **	1.19 × 10^−9^
**Induced labor**	32 (9.88)	292 (90.1)	6 (1.7)	1537 (98.3)	6.5 (3.8–11.0) **	6.05 × 10^−12^
**<Ophthalmic complications>**						
**Cataract**	1 (100%)	0 (0%)	3 (27.3%)	8 (72.7%)	7.3 (0.2–225.9)	0.26
**Glaucoma**	2 (20.0)	8 (80.0)	4 (13.8)	25 (86.2)	1.6 (0.2–10.2)	0.64
**Other**	10 (12.8)	68 (87.2)	28 (8.4)	304 (91.6)	1.6 (0.7–3.4)	0.23

DR: diabetic retinopathy, GDM: gestational diabetes mellitus, pexD: pre-existing diabetes mellitus. Values are presented as the mean ± standard deviation. ** *p* < 0.01.

**Table 3:** The distribution of DR stage from the multicenter cohort.

	N	Age (Years)	NDR	Mild	Moderate	Severe	PDR	Total DR
**pexD**	119	33.4 ± 4.9	88	17	6	4	4	31 (26.1%) *
**GDM**	96	34.3 ± 5.3	94	2	0	0	0	2 (2.1%)
**ODM**	10	30.9 ± 4.6	7	2	1	0	0	3 (30.0%)
**Total**	225	33.6 ± 5.1	189	21	7	4	4	36 (16.0%)

DR: diabetic retinopathy, GDM: gestational diabetes mellitus, NDR: no diabetic retinopathy, ODM: overt diabetes mellitus, pexD: pre-existing diabetes mellitus, PDR: proliferative diabetic retinopathy. Analysis of covariance (ANCOVA) with Tukey-type multiple comparison as a post hoc test was performed. * *p* < 0.05.

**Table 4:** DR progression ratio from multicenter cohort.

	N	Age (Years)	Duration (Years)	DR Progression (%)
**pexD**	102	33.5 ± 4.6	12.9 ± 7.9	10 (9.8)
**GDM**	42	34.1 ± 5.4		0 (0)
**ODM**	5	31.6 ± 3.2		2 (40.0)
**Total**	149	33.6 ± 4.8	12.9 ± 7.9	12 (8.1)

DR: diabetic retinopathy, GDM: gestational diabetes mellitus, ODM: overt diabetes mellitus, pexD: pre-existing diabetes mellitus.
