# Supplementary material for: Trends in the Prevalence and Progression of Diabetic Retinopathy Associated with Hyperglycemic Disorders during Pregnancy in Japan
Source: J Clin Med. 2021 Dec 29;11(1):165. doi: 10.3390/jcm11010165 (PMC8745209; doi:10.3390/jcm11010165)
Supplement: Supplementary file 1 [file jcm-11-00165-s001.zip › jcm11010165 Supplementary Material.pdf]

Supplementary Materials: Trends in the Prevalence and Progression of Diabetic Retinopathy Associated with Hyperglycemic Disorders During Pregnancy in Japan

Masahiko Sugimoto, Kohei Sampa, Hideyuki Tsukitome, Kumiko Kato, Hisashi Matsubara, Shin Asami, Kaori Sekimoto, Shigehiko Kitano, Shigeo Yoshida, Yoshihiro Takamura, Takao Hirano, Toshinori Murata, Miho Shimizu, Takamasa Kinoshita, Sentaro Kusuhara, Osamu Sawada, Masahito Ohji, Rina Yoshikawa, Kazuhiro Kimura, Hiroto Ishikawa, Fumi Gomi, Hiroto Terasaki, Mineo Kondo, Tomoaki Ikeda and on behalf of the Writing Committee of Japan-Clinical Retina STudy Group (J-CREST)†

Table S1. Patients' background data from the multicenter cohort.

	N	Age (years)	Duration (years)	HbA1c (%)	Week	SBP (mmHg)	DBP (mmHg)	Cr (mg/dL)	Insulin (+/−)
**pexD**	119	33.4 ± 4.9	12.2 ± 8.0	6.4 ± 1.2	17.3 ± 6.5	116.6 ± 16.3	69.0 ± 10.9	0.5 ± 0.1	115/4
**GDM**	96	34.3 ± 5.3		5.8 ± 1.1	22.1 ± 7.0	117.8 ± 16.6	71.3 ± 13.0	0.5 ± 0.1	63/33
**ODM**	10	30.9 ± 4.6		7.7 ± 1.1	20.7 ± 9.6	123.8 ± 13.6	72.9 ± 9.7	0.5 ± 0.1	9/1
**Total**	225	33.6 ± 5.1	12.2 ± 8.0	6.3 ± 1.2	19.5 ± 7.1	117.6 ± 16.1	70.0 ± 11.7	0.5 ± 0.1	187/38

Cr: creatinine, DBP: diastolic blood pressure, GDM: gestational diabetes mellitus, ODM: overt diabetes mellitus, pexD: pre-existing diabetes mellitus, SBP: systolic blood pressure.

Table S2. Patients' background data from the multicenter cohort (early and late phase).

	N	Age (Years)	Duration (Years)	HbA1c (%)		SBP (mmHg)		DBP (mmHg)		Cr (mg/dL)		Insulin (+/−)
				Pre	Post	Pre	Post	Pre	Post	Pre	Post	
**pexD**	102	33.5 ± 4.6	12.9 ± 7.9	6.3 ± 1.1	6.4 ± 0.8	114.3 ± 13.6	114.6 ± 13.8	67.3 ± 9.0	69.5 ± 9.7	0.5 ± 0.1	0.5 ± 0.1	100/2
**GDM**	42	34.1 ± 5.4		5.4 ± 0.5	5.6 ± 0.6	116.6 ± 14.2	117.7 ± 11.4	60.7 ± 13.7	69.4 ± 9.2	0.5 ± 0.1	0.5 ± 0.1	27/15
**ODM**	5	31.6 ± 3.2		7.4 ± 0.9	6.1 ± 0.6	120.6 ± 18.9	120.6 ± 12.5	73.0 ± 10.7	75.6 ± 13.0	0.5 ± 0.2	0.6 ± 0.2	4/1
**Total**	149	33.6 ± 4.8	12.9 ± 7.9	6.1 ± 1.1	5.9 ± 0.8	115.3 ± 14.0	115.6 ± 13.2	68.2 ± 9.9	69.7 ± 9.7	0.5 ± 0.1	0.5 ± 0.1	131/18

Cr: creatinine, DBP: diastolic blood pressure, GDM: gestational diabetes mellitus, ODM: overt diabetes mellitus, pexD: pre-existing diabetes mellitus, SBP: systolic blood pressure.

† Writing Committee of Japan—Clinical Retina STudy group (JCREST): Masahiko Sugimoto, Hisashi Matsubara, Kaori Sekimoto, Shigehiko Kitano, Shigeo Yoshida, Yoshihiro Takamura, Takao Hirano, Toshinori Murata, Miho Shimizu, Takamasa Kinoshita, Masahito Ohji, Rina Yoshikawa, Kazuhiro Kimura, Hiroto Ishikawa, Fumi Gomi, Hiroto Terasaki and Mineo Kondo.
